# A Point Prevalence Study of the Provision of Palliative Care for Adult Inpatients With Mental Health Issues

**DOI:** 10.1177/10783903261434242

**Published:** 2026-03-23

**Authors:** Janie Brown, Dipna Martin-Robins, Alannah Cooper

**Affiliations:** 1 Janie Brown, RN, Dip App Sc (Nursing), BN, ICU Cert, Grad Dip AE&T, MEd (Adult), PhD, Curtin School of Nursing, Faculty of Health Sciences, Curtin University, Perth, Western Australia, Australia; St John of God Midland Public and Private Hospitals, Perth, Western Australia, Australia; JBI Evidence Informed Health Care Curtin University, Perth, Western Australia, Australia; 2 Dipna Martin-Robins, RN, BScN, MPC, Royal Perth Hospital, Perth, Western Australia, Australia; 3 Alannah Cooper, RN, BN (Hons), PhD, Murdoch University, Perth, Western Australia, Australia

**Keywords:** mental health, palliative care, point prevalence, hospitals, inpatient

## Abstract

**Background:**

Little is known about the palliative care needs of people living with a mental illness and a life-limiting illness.

**Aims:**

To gain an understanding of palliative care need and service utilization in adult inpatients with mental health issues across a metropolitan area health service in Perth, Western Australia.

**Methods:**

Data were collected at four sites from patient medical records. Adult patients who were admitted at study sites’ mental health units were eligible for inclusion.

**Results:**

In total, 192 patient records were reviewed. Almost one-third of patients (32%, *n* = 61) had at least one condition listed in the Gold Standards Framework, and 30% (*n* = 18) of these could have potentially benefited from palliative care. There was evidence of one patient receiving some form of palliative care.

**Conclusions:**

In this cohort, there was unmet need for palliative care, especially among older adults. The majority of patients with potential palliative care needs were admitted to older adult mental health wards. There are missed opportunities to provide holistic care to adult inpatients with mental health issues experiencing life-limiting conditions, likely to result in poorer symptom control and reduced quality of life. Approaches to identify and respond to palliative care needs in mental health settings need to be adopted.

## Introduction

There is a considerable disparity between the life expectancy of mental health consumers and the general population, due to higher rates of physical illness and premature mortality (Australian Institute of Health and Welfare [AIHW], 2024; Galletly et al., 2016; Lawrence et al., 2013; Olfson et al., 2015). Up to 80% of deaths in people with mental illness are the result of preventable physical health conditions such as cardiovascular disease (CVD), respiratory disease, and cancer (Lawrence et al., 2013). People living with severe mental illness (SMI) are at increased risk of developing CVD, obesity, cardiometabolic comorbidities, and diabetes (Correll et al., 2017; Strassnig et al., 2017). Many factors have been suggested to contribute to the higher rates of physical illness in people with SMI, such as medication side effects, substance misuse, smoking, and sedentary lifestyles (AIHW, 2024; Lawrence & Kisely, 2010).

### Background

Mental and physical health are intertwined, and each is known to affect the other (Doan et al., 2023). Indeed, when mental health and physical health conditions are both present, there are higher rates of morbidity, greater utilization of the resources within the health care system, and poorer quality of life (Doherty & Gaughran, 2014). Addressing physical and mental health needs is essential to providing optimal and holistic patient care. Although there is a body of literature exploring the mental health of people receiving palliative care for a life-limiting illness, little is known about the palliative care needs of people with a mental illness who also experience a life-limiting illness. A life-limiting illness is “an active, progressive, or advanced disease that has little or no prospect of cure,” and the person is likely to die from it in the future (Department of Health, Disability, and Aging, 2025).

Palliative care aims to improve the quality of life and relieve suffering for patients with life-limiting illnesses and their families/caregivers. This care is holistic across the physical, psychological, spiritual, cultural, and social needs of the patient (World Health Organization, 2020). Palliative care is interdisciplinary and can be generalist (basic training) or specialist (complex care requirements) and delivered across all health care settings as well as in the patient’s home (International Association for Hospice and Palliative Care [IAHPC], 2018). Although the terms may be used synonymously, there is a distinction and difference between palliative care (care early in the disease trajectory) and end-of-life care (care of those likely to die within the next 12 months) (Australian Commission on Safety and Quality in Health Care [ACSQHC], 2023; Schüttengruber et al., 2022). Failing to discern and understand this distinction is a barrier to early palliative care (Flierman et al., 2019). However, it is purported that a palliative approach embedded into the care of the person living with a mental illness addresses the expectations of person-centered care, such as autonomy, while improving the quality of their care and the outcomes (Trachsel et al., 2016).

For people living with mental illness, it is more likely that life-limiting physical conditions, e.g., cancer, will be detected later in their trajectory, leading to poorer health outcomes, prolonged time with poor quality of life and burdensome symptoms, and these people are more likely to be referred very late in their disease trajectory, or miss out on palliative care entirely (Sadowska et al., 2023; Shalev et al., 2020). Despite the fact that the mental health workforce are equipped with the necessary communication, assessment, care planning and referral capabilities needed to support the palliative care of people living with a mental and physical illness (Trachsel et al., 2016), psychiatrists and trainees report feeling that they lack the training and clinical skill to support the care of patients and families living with a mental illness (Forster et al., 2017), a factor that may contribute to poorer patient outcomes. A variety of approaches, such as further education and mentorship, may address these deficits (Forster et al., 2017); however, nontraditional (Picot et al., 2015) and interdisciplinary models of care (Lloyd-Williams et al., 2014), tailored to marginalized and vulnerable people with severe and persistent mental illness (Woods et al., 2008) who are also living with a life-limiting illness, may also be beneficial.

Although there are calls and opportunities for further research into palliative care for people living with severe and persistent mental and physical illness (Lloyd-Williams et al., 2014; Woods et al., 2008), it is important that the need for palliative care and prevalence of care provision are established.

This study aimed to explore the palliative care needs and service utilization among adult mental health inpatients within a metropolitan area health service in Perth, Western Australia. The specific objectives were to

1. identify the number and characteristics of adult mental health inpatients across four study sites who had palliative care needs;
2. determine the proportion of these patients currently receiving palliative care or referred to specialist palliative care; and
3. compare referral rates and access to palliative care services across the four sites, each with distinct palliative care models.

## Methods

### Design

This study was part of a broader research initiative and built on earlier investigations into palliative care prevalence across diverse adult populations (Cooper et al., 2021, 2024, 2025). We conducted prospective point prevalence studies at four public hospitals. Data collection occurred on the following dates: Site 4: 24 May 2024, Site 1: 7 June 2024, Site 3: 14 June 2024, and Site 2: 21 June 2024.

### Study Sites

Site 1 is a public hospital comprising 170 medical/surgical beds, 41 mental health beds (with 10 closed at the time of data collection), and a dialysis service. Mental health units include an older adult ward, a locked ward, and two acute wards. Palliative care is provided via a consultative model with 0.2 full-time equivalent (FTE) by a Palliative Medicine Specialist Consultant and 1 FTE across two Nurse Practitioners. In 2023, there were 495 referrals to the palliative care service.

Site 2 is a public hospital with 199 beds, including 100 mental health beds. Other services include rehabilitation and aged care. Mental health services include a youth unit, acute ward, older adult ward, and two locked wards. No formal palliative care service is based on site. However, one Nurse Practitioner from Site 3 provides 0.2 FTE weekly on-site support and phone consultations. Referrals are made by the treating teams.

Site 3 is a larger public hospital with 438 medical/surgical beds and 32 mental health beds across an acute ward, locked ward, and mental health emergency center. The palliative care team includes 1.6 FTE Palliative Medicine Specialists, 1 FTE Registrar, 1.8 FTE Nurse Practitioners, 1 FTE Clinical Nurse, 1 FTE Social Worker, and 1 FTE Secretary. In 2023, there were 1583 referrals to the team.

Site 4, a public-private partnership, includes 196 medical/surgical beds and 56 mental health beds across an acute ward, older adult ward, and locked ward. At the time of data collection, one bed was closed. Palliative care is provided by a 0.5 FTE Palliative Medicine Specialist and 0.8 FTE Clinical Nurse Consultant. In 2023, there were 612 inpatient-only palliative care referrals.

### Sample and Inclusion Criteria

All adult inpatients in mental health units on the data collection day at each site were included. Patients under 18 years old, in emergency departments, medical/surgical wards, or admitted for same-day procedures were excluded.

### Data Collection

Data collection followed a standardized protocol adapted from previous studies (Cooper et al., 2021, 2024, 2025). The study sites used a combination of physical and electronic medical records. The electronic systems included iSoft and BOSSnet. Data were extracted from medical records and entered into Qualtrics. The patient’s age, gender, indigeneity, hospital of admission, and admitting specialty were collected. Trained Registered Nurses assessed records using a data dictionary (Supplementary File 1) to gather data based on the Gold Standards Framework Proactive Identification Guidance (GSF PIG) (Thomas & Armstrong Wilson, 2016).

The Gold Standards Framework Prognostic Indicator Guidance (GSF PIG) is a clinical checklist designed to help health professionals identify patients who may be in the last year of life with potential palliative care needs and who could benefit from a palliative care approach. It includes the “surprise question” (“Would you be surprised if the patient were to die within the next year, months, weeks, or days?”), general indicators of decline and specific indicators for 12 life-limiting conditions: cancer, chronic obstructive pulmonary disease (COPD), dementia, frailty, neurological disease, heart, kidney, and liver disease, motor neuron disease, multiple sclerosis, Parkinson’s disease, and stroke. Criteria for identifying patients who may benefit from palliative care are based on these condition-specific proactive indicators. All entries and information in the medical record were reviewed for the 6 months leading up to each patient’s current admission.

This study used the 2016 version of the GSF PIG, which includes condition-specific criteria. It operates as a binary (yes/no) trigger tool. Meeting the minimum number of clinical indicators suggests that a person may be in the last 12 months of life. If the minimum number of indicators is present, the patient is considered to have a potential palliative care need. The greater the number or severity of indicators present, the stronger the case for initiating or escalating a palliative approach. If these minimum criteria are not met, it is more likely that the person has a life-limiting condition that is progressive in nature, with care needs expected to increase in the future. In a New Zealand acute hospital setting (O’Callaghan et al., 2014), the GSF PIG demonstrated a sensitivity of 62.6% and specificity of 91.9% for predicting 12-month mortality (positive predictive value [PPV] 67.7%, negative predictive value [NPV] 90.0%). In an Australian inpatient cohort (Mudge et al., 2018) using a modified version, including Supportive and Palliative Care Indicators Tool (SPICT) triggers, sensitivity was 78% and specificity was 72% (PPV 38%, NPV 94%).

Assessment of the records focused on physical comorbidities aligned with palliative need, excluding psychosocial concerns like self-harm or suicidal ideation. In this study, palliative care needs were considered met (services accessed) when the treating team applied a palliative care approach, such as holding goals of care discussions with patients or families (Comer et al., 2020), completing advanced care plans to define treatment limits, or referring the patient to specialist palliative care services. This was recorded as a dichotomous yes or no.

### Data Analysis

Data were exported into Stata (StataCorp, 2021). Descriptive statistics, specifically frequencies and percentages, were used to summarize patient characteristics, identify potential palliative care needs, and report service access across the four sites.

### Ethical Considerations

Ethics approval was obtained from two Human Research Ethics Committees (HREC; 5782 & 2122) with a waiver of consent granted for retrospective audit of medical records, in accordance with National Health and Medical Research Council (NHMRC) guidelines (NHMRC, Australian Research Council & Universities Australia, 2018). Reciprocal approval was also obtained from the affiliated university HREC (HRE2023-0339).

### Reporting

This study adheres to the STROBE Statement for observational research. The checklist is provided in Supplementary File 2.

## Results

Of 192 patients’ medical records reviewed across the health service, 75 were from Site 2, 55 from Site 4, and 31 each from Sites 1 and 3.

### Patient Characteristics

**Figure 1.**
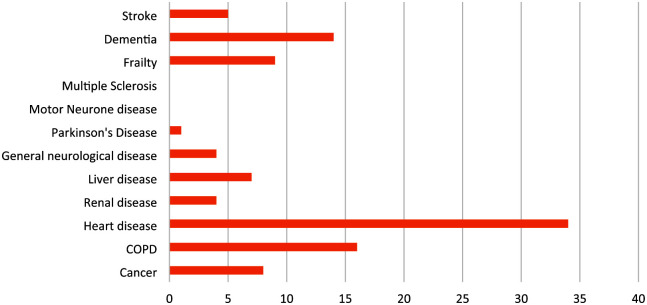
Prevalence of Conditions Listed in the Gold Standards Framework Proactive Identification Guidance.

Among the 192 patients across the health service, 53% (*n* = 101) were male, aged 18–93 (*M* = 50 years). Most were non-Indigenous (90%, *n* = 173); 9% (*n* = 17) identified as Aboriginal, with indigeneity unknown for 1% (*n* = 2) (Table 1). Thirty-two percent (*n* = 61) had a documented life-limiting condition per the GSF PIG (Figure 1).

**Table 1.** Patient Characteristics for All Sites.

	Patient medical records reviewed	Female	Aboriginal or Torres Strait Isander	Age range *(M)*	Patients with a history of ≥1 life-limiting condition in GSF PIG	Patient with a “no” response to surprise question	Patients who may potentially benefit from palliative care
Site 1	31	18	1	20–79 (50)	7	1	2
Site 2	75	31	7	18–93 (54)	37	10	9
Site 3	31	16	4	22–68 (42)	1	1	0
Site 4	55	26	5	18–89 (49)	16	8	7

*Note.* GSF PIG = Gold Standards Framework Proactive Identification Guidance.

At study site 1, among 31 patients, 58% (*n* = 18) were female, aged 20–79 (*M* = 50 years). Almost all were non-Indigenous (97%, *n* = 30); one identified as Aboriginal. Twenty-six percent (*n* = 7) had a life-limiting condition across all ward types (older adult *n* = 3, acute open *n* = 3, and locked ward *n* = 1).

At study site 2, 75 adult records were reviewed (eight <18 years were excluded). Most were male (59%, *n* = 44), aged 18–93 (*M* = 54 years). Nine percent (*n* = 7) identified as Aboriginal.

At study site 3, 31 records were reviewed. Most were female (52%, *n* = 16), aged 22–68 (*M* = 42 years). Thirteen percent (*n* = 4) identified as Aboriginal.

At study site 4, among 55 patients, 53% (*n* = 29) were male, aged 18–89 (*M* = 49 years). Nine percent (*n* = 5) were Aboriginal.

### Palliative Care Need and Service Provision

Among those with a listed condition (*n* = 61) across the health service, 33% (*n* = 20) were identified as not likely to survive the next year (based on the surprise question). Eighteen patients with a listed condition (30%) were assessed as potentially able to benefit from palliative care, representing 9% of all reviewed inpatients. Most of these patients were admitted to older adult mental health wards (83%, *n* = 15). Only one patient across the health service was receiving palliative care (from their treating team); 94% (*n* = 17) had no evidence of referral or care provision.

At study site 1, of the seven patients with listed conditions, one was assessed as likely to die within a year. Two patients (29%) could have potentially benefited from palliative care—both admitted to the older adult ward, and both with no evidence of referral or care provision.

At study site 2, 49% (*n* = 37) of patients had at least one life-limiting condition, particularly among older adult patients (*n* = 19), acute patients (*n* = 8), and patients in the locked ward (*n* = 10). No patients on the youth unit met any criteria. Of the 37 patients, 10 were not expected to live beyond a year. Nine (24%) had potential palliative needs, with six on the older adult ward, two on the acute ward, and one on the locked ward. Only one of these patients was receiving palliative care; the remainder had no documented plans or referrals.

At study site 3, only one patient had a relevant condition and was admitted to the emergency mental health unit. This patient was not identified as nearing the end of life and did not require palliative care.

At study site 4, 16 patients had at least one GSF condition; most were admitted to the older adult (*n* = 11) or acute wards (*n* = 5). Half of these 16 patients were expected to die within the year. Seven (44%) had potential palliative care needs, all on the older adult ward. None were receiving or were referred for palliative care.

## Discussion

This study highlights a significant unmet need for palliative care in adult mental health inpatient settings across a metropolitan health service in Perth, Western Australia. One-third of patients had a life-limiting condition per the GSF PIG, and 30% of those were assessed as potentially benefiting from palliative care. Despite this, only one patient was receiving any palliative care input. The majority of those with potential palliative care needs were older adults, underscoring a critical gap in the integration of palliative approaches within mental health services.

The estimate of adult inpatients with mental health issues and potential palliative care need across the area health service (9%) reported in this study, is lower than our earlier studies in medical and surgical contexts (29%) (Cooper et al., 2021), outpatient (41%) (Cooper et al., 2024) and hemodialysis (45%) (Cooper et al., 2025) contexts. At a hospital level, the highest unmet need in this study is at study site 4 (*n* = 7, 13%), where all of this unmet need is contained in the older adult context. In contrast, there was no unmet need for palliative care at study site 3, where this is no older adult mental health ward and therefore a younger patient cohort.

Unmet need for palliative care in older adults experiencing mental illness represented the majority of unmet need in our cohorts. In a discussion paper exploring older adult mental health patients’ end-of-life and palliative care needs, Kotze and Roos (2022) report that the majority of patients in this cohort have decision-making capacity and should therefore be engaged in palliative care planning. However, the emerging nature of research in this area means that little is known about the palliative care needs or experience of older adult inpatients with mental health issues.

While our findings indicate that the majority of palliative care need is associated with older adult patients, the needs of all adult inpatients with mental health issues should not be neglected. Indeed, we found that almost a quarter of the patients in this study had at least one condition listed in the GSF PIG, but did not require palliative care, but will likely have future needs due to the progressive nature of their conditions. Mental health nurses are well-positioned within the multidisciplinary team to assess for actual and potential palliative care in all adult inpatients with mental health issues, and where indicated, provide basic, generalist palliative care (IAHPC, 2018). If the patient’s care needs are complex, it is appropriate for mental health nurses to escalate their assessment findings to specialist palliative care teams or to the General Practitioner (IAHPC, 2018).

We were unable to find other published prevalence studies exploring palliative care needs in adult inpatients with mental health issues. However, a number of literature reviews (Denduyver et al., 2025; Donald & Stajduhar, 2019; Riley et al., 2022) reflect our findings, reporting that palliative care is being underutilized (Riley et al., 2022), and that the palliative care needs of adult inpatients with mental health issues are suboptimal (Denduyver et al., 2025) or not being met (Donald & Stajduhar, 2019). Other prevalence-type studies explored end-of-life care (Fond et al., 2019) or retrospectively describe the palliative care needs of groups or subgroups of adult patients with a mental illness (Boozalis et al., 2025; Butler & O’Brien, 2018). Although their findings align with ours, comparisons are unable to be made due to the variation in population type (end-of-life, not palliative) or research design (retrospective, not prospective).

### Limitations

We collected data from four sites across a metropolitan area health service in Perth, Western Australia, and as such acknowledge that the generalizability of other findings to other jurisdictions is limited. We caution readers to interpret our findings with their own context in mind. Data were collected at a point in time, and while the data were rigorously collected, we recognize that the data may over- or underrepresent palliative care need; need across the area health service could be different on any given day. The data were collected from the medical records of inpatients, which may have been incomplete at the time of screening.

## Relevance for Clinical Practice

On the basis of this rigorously designed and executed point prevalence study, which provides an estimate of palliative care need for mental health inpatients across a metropolitan area health service in Perth, Western Australia, we make a series of recommendations for clinical practice and practice-based research. It is our intention that these recommendations will guide hospital management teams, clinicians, and researchers to improve access to and delivery of palliative care services in all adult inpatient mental health settings, but with a specific emphasis on older adult contexts. To address unmet need for palliative care, we recommend the following:

- Ongoing palliative care professional development opportunities for all members of the multidisciplinary team, particularly those working in older adult mental health contexts.
- Integration of palliative care principles across mental health contexts.
- Assessment of operational requirements, including staffing levels in palliative care services to meet the level of palliative care needs for adult mental health inpatients.
- Research to identify the barriers to adult mental health inpatient palliative care referral.
- Co-design, development, and testing of an intervention that addresses the barriers to adult mental health inpatient palliative care referral and improves access to palliative care services.

## Conclusion

This study identified potential palliative care needs in patients admitted to inpatient mental health settings, particularly in older adult wards. Of the patients with potential palliative care needs, there was no evidence of any form of palliative care being received in the majority of cases. This points to missed opportunities to provide holistic care to patients and is likely to result in poorer symptom control and a reduced quality of life for patients experiencing life-limiting conditions. Specific approaches to identify and respond to palliative care need in mental health settings need to be adopted.

## Supplemental Material

sj-docx-1-jap-10.1177_10783903261434242 – Supplemental material for A Point Prevalence Study of the Provision of Palliative Care for Adult Inpatients With Mental Health Issues Supplemental material, sj-docx-1-jap-10.1177_10783903261434242 for A Point Prevalence Study of the Provision of Palliative Care for Adult Inpatients With Mental Health Issues by Janie Brown, Dipna Martin-Robins and Alannah Cooper in Journal of the American Psychiatric Nurses Association

sj-docx-2-jap-10.1177_10783903261434242 – Supplemental material for A Point Prevalence Study of the Provision of Palliative Care for Adult Inpatients With Mental Health Issues Supplemental material, sj-docx-2-jap-10.1177_10783903261434242 for A Point Prevalence Study of the Provision of Palliative Care for Adult Inpatients With Mental Health Issues by Janie Brown, Dipna Martin-Robins and Alannah Cooper in Journal of the American Psychiatric Nurses Association

## References

[bibr1-10783903261434242] Australian Commission on Safety and Quality in Health Care. (2023). National consensus statement: Essential elements for safe and high-quality end-of-life care. ACSQHC. https://www.safetyandquality.gov.au/sites/default/files/2023-12/national_consensus_statement_-_essential_elements_for_safe_and_high-quality_end-of-life_care.pdf

[bibr2-10783903261434242] Australian Institute of Health and Welfare. (2024). Physical health of people with mental illness. https://www.aihw.gov.au/reports/physical-health-of-people-with-mental-illness

[bibr3-10783903261434242] Boozalis J. Perreault J. Turner H. I. Wu W. C. Browne J. Jiang L. Wice M. Rudolph J. L. Stafford J. P. (2025). A retrospective study of deceased veterans with serious mental illness and heart failure: Analysis of palliative care and mental health collaboration on hospice utilization. General Hospital Psychiatry, 96, 151–155. 10.1016/j.genhosppsych.2025.07.009 40674777

[bibr4-10783903261434242] Butler H. O’Brien A. J. (2018). Access to specialist palliative care services by people with severe and persistent mental illness: A retrospective cohort study. International Journal of Mental Health Nursing, 27(2), 737–746. https://doi.org/https://doi.org/10.1111/inm.12360 28692186 10.1111/inm.12360

[bibr5-10783903261434242] Comer A. Fettig L. Torke A. M. (2020). Identifying goals of care. Medical Clinics of North America, 104(5), 767–775. 10.1016/j.mcna.2020.06.002 32773044 PMC7458156

[bibr6-10783903261434242] Cooper A. L. Mazzer J. Martin-Robins D. Brown J. A. (2021). A point prevalence study of palliative care need and referral rates in adult inpatients. Journal of Clinical Nursing, 31(21–22), 3144–3154. 10.1111/jocn.16148 34850483

[bibr7-10783903261434242] Cooper A. L. Panizza N. Bartlett R. Martin-Roberts D. Brown J. A. (2025). A period prevalence study of palliative care need and provision in adult patients attending hospital-based dialysis units. Journal of Nephrology, 38, 687–695. 10.1007/s40620-024-02193-2 39869144 PMC11961506

[bibr8-10783903261434242] Cooper A. L. Tucknott S. Mazzer J. Martin-Robins D. Brown J. A. (2024). A period prevalence study of palliative care need in adult outpatients. Progress in Palliative Nursing, 32(3), 176–182. 10.1080/09699260.2024.2339095

[bibr9-10783903261434242] Correll C. U. Solmi M. Veronese N. Bortolato B. Rosson S. Santonastaso P. Thapa-Chhetri N. Fornaro M. Gallicchio D. Collantoni E. Pigato G. Favaro A. Monaco F. Kohler C. Vancampfort D. Ward P. B. Gaughran F. Carvalho A. F. Stubbs B. (2017). Prevalence, incidence and mortality from cardiovascular disease in patients with pooled and specific severe mental illness: A large-scale meta-analysis of 3,211,768 patients and 113,383,368 controls. World Psychiatry, 16(2), 163–180. 10.1002/wps.20420 28498599 PMC5428179

[bibr10-10783903261434242] Denduyver J. Detraux J. Weydts J. De Hert M. (2025). End-of-life care for people with severe and persistent mental illness and a life-limiting disease: An umbrella review. European Psychiatry, 68(1), e49. 10.1192/j.eurpsy.2025.2440 PMC12041735 40123415

[bibr11-10783903261434242] Department of Health, Disability, and Aging. (2025). What is palliative care? Commonwealth of Australia. https://www.health.gov.au/topics/palliative-care/about-palliative-care/what-is-palliative-care#:~:text=A%20life%2Dlimiting%20illness%20is,for%20many%20years%20to%20come

[bibr12-10783903261434242] Doan T. Ha V. Strazdins L. Chateau D. (2023). Healthy minds live in healthy bodies—effect of physical health on mental health: Evidence from Australian longitudinal data. Current Psychology, 42, 18702–18713. 10.1007/s12144-022-03053-7

[bibr13-10783903261434242] Doherty A. M. Gaughran F. (2014). The interface of physical and mental health. Social Psychiatry and Psychiatric Epidemiology, 49(5), 673–682. 10.1007/s00127-014-0847-7 24562320

[bibr14-10783903261434242] Donald E. E. Stajduhar K. I. (2019). A scoping review of palliative care for persons with severe persistent mental illness. Palliative and Supportive Care, 17(4), 479–487. 10.1017/S1478951519000087 30887934

[bibr15-10783903261434242] Flierman I. Nugteren I. C. van Seben R. Buurman B. M. Willems D. L. (2019). How do hospital-based nurses and physicians identify the palliative phase in their patients and what difficulties exist? A qualitative interview study. BMC Palliative Care, 18(1), 54. 10.1186/s12904-019-0439-0 31288816 PMC6617645

[bibr16-10783903261434242] Fond G. Salas S. Pauly V. Baumstarck K. Bernard C. Orleans V. Llorca P.-M. Lancon C. Auquier P. Boyer L. (2019). End-of-life care among patients with schizophrenia and cancer: A population-based cohort study from the French national hospital database End-of-life care among patients with schizophrenia and cancer: A population-based cohort study from the French national hospital database. The Lancet. *Public Health*, 4(11), e583–e591. 10.1016/S2468-2667(19)30187-2 31677777

[bibr17-10783903261434242] Forster B. C. Proskurin H. Kelly B. Lovell M. R. Ilchef R. Clayton J. M. (2017). Psychiatry trainees’ views and educational needs regarding the care of patients with a life-limiting illness. Palliative and Supportive Care, 15(2), 231–241. 10.1017/s1478951516000365 27320847

[bibr18-10783903261434242] Galletly C. Castle D. Dark F. Humberstone V. Jablensky A. Killackey E. Kulkarni J. McGorry P. Nielssen O. Tran N. (2016). Royal Australian and New Zealand College of Psychiatrists clinical practice guidelines for the management of schizophrenia and related disorders. Australian & New Zealand Journal of Psychiatry, 50(5), 410–472. 10.1177/0004867416641195 27106681

[bibr19-10783903261434242] The International Association for Hospice and Palliative Care. (2018). Global Consensus based palliative care definition. https://iahpc.org/what-we-do/research/consensus-based-definition-of-palliative-care/definition/

[bibr20-10783903261434242] Kotze C. Roos J. L. (2022). Ageism, human rights and ethical aspects of end-of-life care for older people with serious mental illness. Frontiers in Psychiatry, 13, Article 906873. 10.3389/fpsyt.2022.906873 PMC9366006 35966471

[bibr21-10783903261434242] Lawrence D. Hancock K. J. Kisely S. (2013). The gap in life expectancy from preventable physical illness in psychiatric patients in Western Australia: Retrospective analysis of population based registers. British Medical Journal, 346(7909), f2539. 10.1136/bmj.f2539 PMC3660620 23694688

[bibr22-10783903261434242] Lawrence D. Kisely S. (2010). Inequalities in healthcare provision for people with severe mental illness. Journal of Psychopharmacology, 24(Suppl. 4), 61–68. 10.1177/1359786810382058 20923921 PMC2951586

[bibr23-10783903261434242] Lloyd-Williams M. Abba K. Crowther J. (2014). Supportive and palliative care for patients with chronic mental illness including dementia. Current Opinion in Supportive and Palliative Care, 8(3), 303–307. 10.1097/spc.0000000000000064 25004172

[bibr24-10783903261434242] Mudge A. M. Douglas C. Sansome X. Tresillian M. Murray S. Finnigan S. Blaber C. R. (2018). Risk of 12-month mortality among hospital inpatients using the surprise question and SPICT criteria: A prospective study. BMJ Supportive & Palliative Care, 8(2), 213. 10.1136/bmjspcare-2017-001441 29500239

[bibr25-10783903261434242] National Health and Medical Research Council, Australian Research Council & Universities Australia. (2018). Australian code for the responsible conduct of research. Commonwealth of Australia. https://www.nhmrc.gov.au/about-us/publications/australian-code-responsible-conduct-research-2018#block-views-block-file-attachments-content-block-1

[bibr26-10783903261434242] O’Callaghan A. Laking G. Frey R. Robinson J. Gott M. (2014). Can we predict which hospitalised patients are in their last year of life? A prospective cross-sectional study of the Gold Standards Framework Prognostic Indicator Guidance as a screening tool in the acute hospital setting. Palliative Medicine, 28(8), 1046–1052. 10.1177/0269216314536089 24854032

[bibr27-10783903261434242] Olfson M. Gerhard T. Huang C. Crystal S. Stroup T. S. (2015). Premature mortality among adults with schizophrenia in the United States. JAMA Psychiatry, 72(12), 1172–1181. 10.1001/jamapsychiatry.2015.1737 26509694

[bibr28-10783903261434242] Picot S. A. Glaetzer K. M. Myhill K. J. (2015). Coordinating end of life care for individuals with a mental illness: A nurse practitioner collaboration. Collegian, 22(1), 143–149. 10.1016/j.colegn.2013.12.007 26285419

[bibr29-10783903261434242] Riley K. Hupcey J. E. Kowalchik K. (2022). Palliative care in severe and persistent mental illness. Journal of Hospice and Palliative Nursing: JHPN, 24(3), E88–E93. 10.1097/NJH.0000000000000855 35285463

[bibr30-10783903261434242] Sadowska K. Fong T. Horning D. R. McAteer S. Ekwebelem M. I. Demetres M. Reid M. C. Shalev D. (2023). Psychiatric comorbidities and outcomes in palliative and end-of-life care: A systematic review. Journal of Pain and Symptom Management, 66(1), e129–e151. 10.1016/j.jpainsymman.2023.03.007 PMC10330030 37003308

[bibr31-10783903261434242] Schüttengruber G. Großschädl F. Lohrmann C. (2022). A consensus definition of end of life from an international and interdisciplinary perspective: A delphi panel study. Journal of Palliative Medicine, 25(11), 1677–1685. 10.1089/jpm.2022.0030 35549439

[bibr32-10783903261434242] Shalev D. Fields L. Shapiro P. A. (2020). End-of-life care in individuals with serious mental illness. Psychosomatics, 61(5), 428–435. 10.1016/j.psym.2020.06.003 32660874 PMC7290196

[bibr33-10783903261434242] StataCorp. (2021). Stata: Release 17 [Statistical Software]. StataCorp LLC.

[bibr34-10783903261434242] Strassnig M. Kotov R. Cornaccio D. Fochtmann L. Harvey P. D. Bromet E. J. (2017). Twenty-year progression of body mass index in a county-wide cohort of people with schizophrenia and bipolar disorder identified at their first episode of psychosis. Bipolar Disorders, 19(5), 336–343. 10.1111/bdi.12505 28574189 PMC5568920

[bibr35-10783903261434242] Thomas K. T. Armstrong Wilson J. (2016). The Gold Standards Framework proactive identification guidance (6th ed.). Royal College of General Practitioners.

[bibr36-10783903261434242] Trachsel M. Irwin S. A. Biller-Andorno N. Hoff P. Riese F. (2016). Palliative psychiatry for severe persistent mental illness as a new approach to psychiatry? Definition, scope, benefits, and risks. BMC Psychiatry, 16, Article 260 10.1186/s12888-016-0970-y PMC4957930 27450328

[bibr37-10783903261434242] Woods A. Willison K. Kington C. Gavin A. (2008). Palliative care for people with severe persistent mental illness: A review of the literature. Canadian Journal of Psychiatry, 53(11), 725–736. 10.1177/070674370805301104 19087466

[bibr38-10783903261434242] World Health Organization. (2020). Palliative care. https://www.who.int/news-room/fact-sheets/detail/palliative-care

